# Supplementary material for: Misfolding of Lysosomal α-Galactosidase a in a Fly Model and Its Alleviation by the Pharmacological Chaperone Migalastat
Source: Int J Mol Sci. 2020 Oct 7;21(19):7397. doi: 10.3390/ijms21197397 (PMC7583893; doi:10.3390/ijms21197397)
Supplement: Supplementary file 1 [file ijms-21-07397-s001.pdf]

Supplementary figure

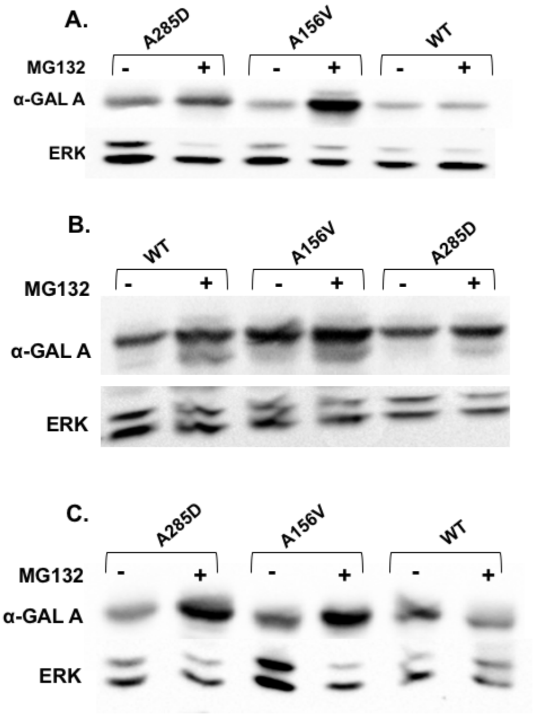

Figure S1. Mutant α-Gal A undergoes ERAD. **A, B. C.** Twenty-four hours after transfection of HEK293T cells with pcDNA4-MycHis-α-Gal A expressing plasmids (human WT, A156V, A285D), they were treated with 25 μM MG132 for 20 h, and their lysates were subjected to Western blotting and interaction with anti-Myc antibody, to identify α-Gal A with and anti ERK antibodies, as a loading control. To quantify the results, α-Gal A intensity in each lane was divided by that of ERK in the same lane, and the number obtained for every variant without treatment was considered to be 1. The results shown in Fig. 1F are the mean ± SEM of the three presented experiments.
